# 6-Fluoro-1,3,4-triphenyl-1*H*-pyrazolo[3,4-*b*]quinoline benzene hemisolvate

**DOI:** 10.1107/S1600536810004496

**Published:** 2010-02-10

**Authors:** Paweł Szlachcic, Katarzyna Stadnicka

**Affiliations:** a Department of Chemistry and Physics, Agricultural University, 30-149 Kraków, Poland; b Faculty of Chemistry, Jagiellonian University, 30-060 Kraków, Poland

## Abstract

In the title compound, C_28_H_18_FN_3_·0.5C_6_H_6_, the 1*H*-pyrazolo[3,4-*b*]quinoline core is almost planar (r.m.s = 0.0371 Å, maximum deviation = 0.0571 Å) and aromatic. The solvent benzene mol­ecules are located around inversion centres. In the crystal, mol­ecules related by centres of symmetry form dimers, with distances of 3.932 (3) Å between best planes through the fused core due to π⋯π stacking. The phenyl substituents at positions 1, 3 and 4, are twisted away from the core, making dihedral angles of 29.66 (7), 44.59 (7) and 67.94 (6)°, respectively.

## Related literature

For the synthesis of 1*H*-pyrazolo[3,4-*b*]quinoline derivatives, see: Chaczatrian *et al.* (2003 ▶, 2007 ▶). For their photophysical properties, see: Gondek *et al.* (2006 ▶). For the use of a fluorine derivative of 1*H*-pyrazolo[3,4-*b*]quinoline in organic light-emitting diode preparation, see: Tao *et al.* (2001 ▶). For the effect of substituents on aromatic ring geometry, see: Domen­icano *et al.* (1975 ▶).
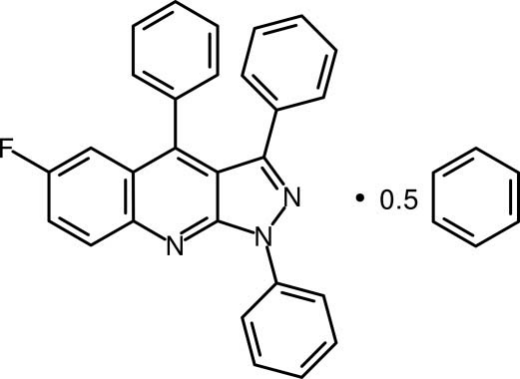


## Experimental

### 

#### Crystal data

C_28_H_18_FN_3_·0.5C_6_H_6_
*M* *_r_* = 454.51
Monoclinic, 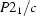

*a* = 13.2941 (4) Å
*b* = 9.7419 (3) Å
*c* = 20.7608 (5) Å
β = 118.559 (2)°
*V* = 2361.58 (12) Å^3^
*Z* = 4
Mo *K*α radiation
μ = 0.08 mm^−1^
*T* = 293 K
0.40 × 0.25 × 0.03 mm

#### Data collection

Nonius KappaCCD diffractometer
Absorption correction: multi-scan (*HKL* *DENZO* and *SCALEPACK*; Otwinowski & Minor, 1997 ▶) *T*_min_ = 0.968, *T*_max_ = 0.998
8615 measured reflections
5135 independent reflections
2974 reflections with *I* > 2σ(*I*)
*R*_int_ = 0.051

#### Refinement

*R*[*F* ^2^ > 2σ(*F* ^2^)] = 0.064
*wR*(*F* ^2^) = 0.137
*S* = 1.06
5135 reflections
317 parameters
3 restraints
H-atom parameters constrained
Δρ_max_ = 0.18 e Å^−3^
Δρ_min_ = −0.19 e Å^−3^

### 

Data collection: *COLLECT* (Nonius, 1998 ▶); cell refinement: *HKL* *SCALEPACK* (Otwinowski & Minor, 1997 ▶); data reduction: *HKL* *DENZO* and *SCALEPACK*; program(s) used to solve structure: *SIR92* (Altomare *et al.*, 1994 ▶); program(s) used to refine structure: *SHELXL97* (Sheldrick, 2008 ▶); molecular graphics: *ORTEP-3* (Farrugia, 1997 ▶); software used to prepare material for publication: *SHELXL97*.

## Supplementary Material

Crystal structure: contains datablocks global, I. DOI: 10.1107/S1600536810004496/gk2254sup1.cif

Structure factors: contains datablocks I. DOI: 10.1107/S1600536810004496/gk2254Isup2.hkl

Additional supplementary materials:  crystallographic information; 3D view; checkCIF report

## Figures and Tables

**Fig. 1.**
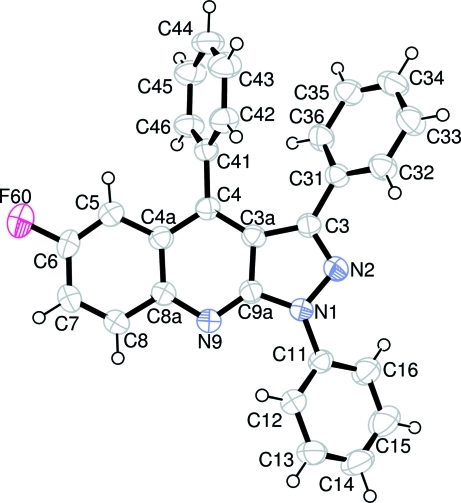
The conformation of the 6-fluoro-1,3,4-triphenyl-1H-pyrazolo[3,4-b]quinoline molecule with the atom numbering scheme. Displacement ellipsoids are drawn at the 50% probability level and H atoms are shown as small spheres of arbitrary radii.

**Fig. 2.**
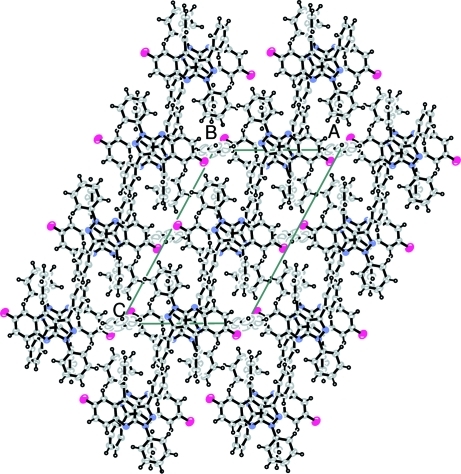
The unit cell contents of the title compound in projection along [010]. The solvent molecules (benzene) are present at the centres of symmetry 0,0,0 and 0,1/2,1/2.

## supplementary crystallographic information

### Comment

Some of the 1*H*-pyrazolo[3,4-*b*]quinoline (PQ) derivatives containing hydrogen, phenyl or methyl substituents, and their combination, showed interesting photophysical properties (Gondek *et al.*, 2006). A relatively high quantum efficiency allowed to propose the investigated materials' blue-light luminophore. The approach was recommended for searching the organic chromophore for organic light-emitting diodes (OLED). To synthesize different sort of PQ derivatives, a new method of preparation developed in the research group led by professor Tomasik (Chaczatrian *et al.*, 2003, Chaczatrian *et al.*, 2007) can be used.

It is known that in the case of 6-fluoro-1-methyl-3-phenyl-1*H*-pyrazolo[3,4-*b*]quinoline, the incorporation of fluorine atom into PQ molecule rises the values of HOMO/LUMO and ionisation potential of the luminophore in comparison to PQ itself (Tao *et al.*, 2001). Because of that, the method of synthesis mentioned above was used for the preparation of a series of compounds containing fluorine substituents to make it useful for construction of OLED cells with Mg/Ag alloy cathode or even Al cathode.

The promising results of using the PQ flurorine derivatives for OLED preparation will be published elsewhere.

The shape of the title molecule is shown in Fig. 1. The core of the molecule, 1*H*-pyrazolo[3,4-*b*]quinoline, is planar and aromatic. All three phenyl substituents' planes are twisted against the core moiety with the torsion angles N2—N1—C11—C16 = -30.1 (3), N2—C3—C31—C32 = -41.7 (3) and C3a—C4—C41—C42 = -66.5 (3)°. For the fluorine substituent at C6, the enlargement of the endocyclic C5—C6—C7 angle, up to 124.0 (3), is caused by σ-electron-withdrawing effect of the fluorine atom (Domenicano *et al.* 1975).

The packing of the molecules (Fig. 2) is determined by intermolecular π···π stacking. The molecules realted by the centres of symmetry at 1/2, 0, 1/2 and 0, 1/2, 0 (*P*2_1_/c, position 2 c -1) form dimers due to this type of interaction. The shortest distances were found between the rings N1—N2—C3—C3a—C9a and C4a—C5—C6—C7—C8, at 1-x, -y, 1-z, for which the centre of gravity distance is 4.038 (3) Å, as well as for the rings C3a—C4—C4a—C8a—N9—C9a and C3a—C4—C4a—C8a—N9—C9a, at 1-x, -y, 1-z, for which the centre of gravity distance is 3.964 (3) Å). The distance between the best planes of the whole aromatic core was found to be 3.932 (3) Å.

The structure is stabilized by several C—H···π interactions with the geometry parameters (H···A /Å, D···A /Å, <DHA /°, respectively) given below.

C32—H32···Cg(C3a—C4—C4a—C8a—N9—C9a at 1-x, 1-y, 1-z): 3.017, 3.621, 124;

C43—H43···Cg(C11—C12—C13—C14—C15—C16 at 1-x, 1-y, 1-z): 2.676, 3.567, 161;

C46—H46···Cg(C11—C12—C13—C14—C15—C16 at 1-x, -y, 1-z): 2.878, 3.656, 142;

C15—H15···Cg(C41—C42—C43—C44—C45—C46 at x-1, -y+1/2, z-1/2): 2.887, 3.817, 180.

The molecules of the title compound co-crystallize with the benzene molecules in the ratio 2:1. The benzene molecules occupy the centres of symmetry of the position 2 a (space group *P*2_1_/c): 0,0,0 and 0,1/2,1/2.

### Experimental

The title compound was synthesized using procedure already described in literature (Chaczatrian *et al.*, 2003, Chaczatrian *et al.*, 2007) from 4-fluoroaniline, benzaldehyde and 1,3-diphenyl-4,5-dihydro-1*H*-pyrazol-5-one (15 mmol of each substrate, ethylene glycol as a solvent). The product was purified by flash chromatography on Al_2_O_3_ with chloroform as a solvent, followed by crystallization from toluene to give 1.00 g (16% yield) of yellow crystalline solid, mp. 466.5-468.5 K. ^1^H NMR (CDCl_3_): δ 7.03-7.09 (m, 2H), 7.12-7.23 (m, 7H), 7.30-7.36 (m, 2H), 7.50 (ddd, *J* = 10.5, 2.9, 0.6 Hz, 1H) 7.53-7.61 (m, 3H), 8.24 (ddd, *J* = 9.2, 5.4, 0.6 Hz, 1H), 8.57-8.60 (m, 2H); ^13^C NMR (CDCl_3_): δ 109.5 (d, *J*_CF_ = 23.3 Hz), 115.2, 120.9, 121.4 (d, *J*_CF_ = 27.0 Hz), 123.8 (d, *J*_CF_ = 9.3 Hz), 125.3, 125.5, 127.5, 127.7, 128.1, 128.2, 128.6, 129.0, 130.2, 131.4 (d, *J*_CF_ = 8.9 Hz), 132.4, 134.2, 139.8, 145.6, 146.3, 150.1, 159.0 (d, *J*_CF_ = 245.2 Hz); ^19^F NMR (CDCl_3_): δ -118.5. Single crystals suitable for X-ray diffraction were grown by slow evaporation from benzene solution.

### Refinement

H atoms were included into refinement in geometrically calculated positions, with C—H = 0.93 Å, and *U*_iso_(H) = 1.2*U*_eq_(parent C) for the aromatic CH groups, and constrained as a part of a riding model. The distances between carbon atoms of the benzene ring of the solvent molecule were constrained to 1.397 (4) Å, using DFIX procedure (*SHELXL-97*; Sheldrick, 2008). The resulting C—C distances of the benzene ring are smaller probably due to large thermal motion or static disorder.

### Crystal data

**Table:**

C_28_H_18_FN_3_·0.5C_6_H_6_	*F*(000) = 948
*M**_r_* = 454.51	*D*_x_ = 1.278 Mg m^−^^3^
Monoclinic, *P*2_1_/*c*	Melting point = 466.5–468.5 K
Hall symbol: -P 2ybc	Mo *K*α radiation, λ = 0.71073 Å
*a* = 13.2941 (4) Å	Cell parameters from 5207 reflections
*b* = 9.7419 (3) Å	θ = 1.0–27.5°
*c* = 20.7608 (5) Å	µ = 0.08 mm^−^^1^
β = 118.559 (2)°	*T* = 293 K
*V* = 2361.58 (12) Å^3^	Plate, yellow
*Z* = 4	0.40 × 0.25 × 0.03 mm

### Data collection

**Table:**

Nonius KappaCCD diffractometer	5135 independent reflections
Radiation source: fine-focus sealed tube	2974 reflections with *I* > 2σ(*I*)
horizontally mounted graphite crystal	*R*_int_ = 0.051
Detector resolution: 9 pixels mm^-1^	θ_max_ = 27.0°, θ_min_ = 3.1°
ω scans at χ = 55 °	*h* = −16→16
Absorption correction: multi-scan (*HKL**DENZO* and *SCALEPACK*; Otwinowski & Minor, 1997)	*k* = −10→12
*T*_min_ = 0.968, *T*_max_ = 0.998	*l* = −26→26
8615 measured reflections	

### Refinement

**Table:**

Refinement on *F*^2^	Secondary atom site location: difference Fourier map
Least-squares matrix: full	Hydrogen site location: inferred from neighbouring sites
*R*[*F*^2^ > 2σ(*F*^2^)] = 0.064	H-atom parameters constrained
*wR*(*F*^2^) = 0.137	*w* = 1/[σ^2^(*F*_o_^2^) + (0.0426*P*)^2^ + 0.742*P*] where *P* = (*F*_o_^2^ + 2*F*_c_^2^)/3
*S* = 1.06	(Δ/σ)_max_ < 0.001
5135 reflections	Δρ_max_ = 0.18 e Å^−^^3^
317 parameters	Δρ_min_ = −0.19 e Å^−^^3^
3 restraints	Extinction correction: *SHELXL*
0 constraints	Extinction coefficient: 0.0045 (8)
Primary atom site location: structure-invariant direct methods	

### Special details

**Table:**

Refinement. Refinement of *F*^2^ against all reflections. The weighted *R*-factor wR and goodness of fit *S* are based on *F*^2^, conventional *R*-factors *R* are based on *F*, with *F* set to zero for negative *F*^2^. The threshold expression of *F*^2^ > σ(*F*^2^) is used only for calculating *R*-factors(gt) etc. and is not relevant to the choice of reflections for refinement. *R*-factors based on *F*^2^ are statistically about twice as large as those based on *F*, and *R*- factors based on all data will be even larger.

### Fractional atomic coordinates and isotropic or equivalent isotropic displacement parameters (Å^2^)

**Table:**

	*x*	*y*	*z*	*U*_iso_*/*U*_eq_	
N1	0.36548 (14)	0.2474 (2)	0.40940 (9)	0.0447 (5)	
N2	0.34691 (15)	0.3176 (2)	0.46036 (9)	0.0468 (5)	
C3	0.44144 (18)	0.3100 (2)	0.52398 (11)	0.0404 (5)	
C3A	0.52804 (17)	0.2324 (2)	0.51683 (10)	0.0383 (5)	
C4	0.64187 (17)	0.1959 (2)	0.56111 (11)	0.0378 (5)	
C4A	0.69378 (17)	0.1174 (2)	0.52705 (11)	0.0410 (5)	
C5	0.81112 (19)	0.0783 (3)	0.56551 (12)	0.0505 (6)	
H5	0.8560	0.1013	0.6146	0.061*	
C6	0.85632 (19)	0.0074 (3)	0.52978 (13)	0.0580 (7)	
C7	0.7950 (2)	−0.0309 (3)	0.45635 (13)	0.0615 (7)	
H7	0.8301	−0.0797	0.4340	0.074*	
C8	0.6834 (2)	0.0046 (3)	0.41845 (12)	0.0545 (6)	
H8	0.6412	−0.0215	0.3696	0.065*	
C8A	0.62881 (18)	0.0809 (2)	0.45139 (11)	0.0427 (5)	
N9	0.51761 (14)	0.1184 (2)	0.40778 (9)	0.0446 (5)	
C9A	0.47403 (17)	0.1924 (2)	0.44168 (11)	0.0395 (5)	
C11	0.27483 (18)	0.2366 (2)	0.33624 (11)	0.0419 (5)	
C12	0.2993 (2)	0.2270 (2)	0.27896 (12)	0.0511 (6)	
H12	0.3748	0.2300	0.2878	0.061*	
C13	0.2108 (2)	0.2130 (3)	0.20837 (12)	0.0634 (7)	
H13	0.2272	0.2043	0.1697	0.076*	
C14	0.0998 (2)	0.2118 (3)	0.19449 (15)	0.0734 (8)	
H14	0.0406	0.2027	0.1467	0.088*	
C15	0.0762 (2)	0.2241 (3)	0.25178 (15)	0.0757 (9)	
H15	0.0004	0.2250	0.2424	0.091*	
C16	0.16303 (19)	0.2353 (3)	0.32298 (13)	0.0592 (7)	
H16	0.1463	0.2418	0.3616	0.071*	
C31	0.44317 (17)	0.3780 (2)	0.58814 (11)	0.0401 (5)	
C32	0.39465 (19)	0.5068 (3)	0.58066 (12)	0.0503 (6)	
H32	0.3634	0.5516	0.5357	0.060*	
C33	0.3925 (2)	0.5690 (3)	0.63981 (14)	0.0606 (7)	
H33	0.3587	0.6548	0.6343	0.073*	
C34	0.4402 (2)	0.5045 (3)	0.70678 (13)	0.0613 (7)	
H34	0.4390	0.5469	0.7466	0.074*	
C35	0.4892 (2)	0.3785 (3)	0.71493 (12)	0.0566 (7)	
H35	0.5221	0.3356	0.7605	0.068*	
C36	0.49028 (19)	0.3141 (3)	0.65598 (12)	0.0493 (6)	
H36	0.5228	0.2274	0.6619	0.059*	
C41	0.70800 (17)	0.2372 (2)	0.63992 (11)	0.0387 (5)	
C42	0.73378 (19)	0.3734 (2)	0.65922 (12)	0.0487 (6)	
H42	0.7129	0.4401	0.6230	0.058*	
C43	0.7907 (2)	0.4104 (3)	0.73242 (13)	0.0608 (7)	
H43	0.8088	0.5020	0.7454	0.073*	
C44	0.8207 (2)	0.3122 (3)	0.78626 (13)	0.0611 (7)	
H44	0.8572	0.3379	0.8354	0.073*	
C45	0.7969 (2)	0.1768 (3)	0.76749 (12)	0.0582 (7)	
H45	0.8181	0.1104	0.8039	0.070*	
C46	0.74159 (19)	0.1389 (2)	0.69457 (11)	0.0499 (6)	
H46	0.7267	0.0466	0.6820	0.060*	
F60	0.96931 (12)	−0.0276 (2)	0.56686 (9)	0.0955 (6)	
C101	1.0871 (4)	−0.0806 (9)	1.0023 (3)	0.1389 (18)	
H101	1.1454	−0.1349	1.0034	0.167*	
C102	1.0815 (5)	0.0560 (10)	0.9865 (3)	0.1379 (19)	
H102	1.1373	0.0954	0.9775	0.165*	
C103	0.9946 (7)	0.1359 (5)	0.9837 (2)	0.1363 (19)	
H103	0.9914	0.2286	0.9721	0.164*	

### Atomic displacement parameters (Å^2^)

**Table:**

	*U*^11^	*U*^22^	*U*^33^	*U*^12^	*U*^13^	*U*^23^
N1	0.0421 (10)	0.0571 (13)	0.0335 (10)	0.0085 (10)	0.0169 (9)	−0.0020 (9)
N2	0.0485 (11)	0.0540 (13)	0.0401 (11)	0.0080 (10)	0.0229 (9)	0.0002 (9)
C3	0.0440 (12)	0.0432 (14)	0.0358 (12)	0.0044 (11)	0.0206 (11)	0.0056 (10)
C3A	0.0453 (13)	0.0368 (13)	0.0351 (12)	0.0007 (11)	0.0211 (10)	0.0013 (9)
C4	0.0414 (12)	0.0349 (12)	0.0353 (11)	0.0003 (11)	0.0168 (10)	0.0037 (9)
C4A	0.0416 (12)	0.0418 (13)	0.0380 (12)	0.0011 (11)	0.0177 (10)	0.0010 (10)
C5	0.0463 (13)	0.0584 (16)	0.0404 (13)	0.0085 (13)	0.0156 (11)	0.0008 (11)
C6	0.0400 (13)	0.0725 (19)	0.0547 (15)	0.0177 (14)	0.0172 (12)	0.0041 (14)
C7	0.0570 (16)	0.075 (2)	0.0553 (16)	0.0186 (14)	0.0290 (13)	−0.0066 (14)
C8	0.0532 (14)	0.0639 (17)	0.0440 (13)	0.0084 (14)	0.0212 (12)	−0.0091 (12)
C8A	0.0431 (13)	0.0456 (14)	0.0401 (12)	0.0012 (11)	0.0206 (11)	−0.0003 (10)
N9	0.0413 (11)	0.0514 (12)	0.0384 (10)	0.0032 (10)	0.0169 (9)	−0.0041 (9)
C9A	0.0402 (12)	0.0412 (13)	0.0377 (12)	0.0035 (11)	0.0191 (10)	0.0020 (10)
C11	0.0436 (12)	0.0401 (13)	0.0361 (12)	0.0040 (11)	0.0142 (10)	0.0022 (10)
C12	0.0479 (13)	0.0594 (17)	0.0409 (13)	0.0093 (12)	0.0172 (11)	0.0006 (11)
C13	0.0744 (18)	0.0673 (19)	0.0382 (14)	0.0158 (15)	0.0187 (13)	−0.0009 (12)
C14	0.0589 (17)	0.082 (2)	0.0484 (16)	0.0022 (16)	0.0005 (14)	−0.0086 (14)
C15	0.0429 (14)	0.102 (3)	0.0670 (19)	−0.0061 (15)	0.0139 (14)	−0.0044 (17)
C16	0.0460 (14)	0.077 (2)	0.0506 (15)	−0.0011 (14)	0.0199 (12)	0.0026 (13)
C31	0.0411 (12)	0.0450 (14)	0.0372 (12)	−0.0015 (11)	0.0210 (10)	−0.0006 (10)
C32	0.0610 (15)	0.0487 (15)	0.0438 (13)	0.0026 (13)	0.0270 (12)	0.0016 (12)
C33	0.0756 (17)	0.0479 (16)	0.0621 (17)	0.0063 (14)	0.0361 (14)	−0.0061 (13)
C34	0.0718 (17)	0.0712 (19)	0.0493 (15)	−0.0060 (16)	0.0357 (13)	−0.0152 (14)
C35	0.0596 (15)	0.075 (2)	0.0414 (13)	0.0014 (15)	0.0291 (12)	0.0023 (13)
C36	0.0556 (14)	0.0539 (16)	0.0455 (13)	0.0076 (13)	0.0300 (11)	0.0070 (12)
C41	0.0404 (12)	0.0406 (13)	0.0345 (11)	0.0021 (11)	0.0174 (10)	0.0011 (10)
C42	0.0553 (14)	0.0399 (14)	0.0430 (13)	0.0013 (12)	0.0172 (11)	0.0041 (11)
C43	0.0685 (17)	0.0440 (15)	0.0550 (16)	−0.0071 (14)	0.0175 (14)	−0.0106 (13)
C44	0.0661 (16)	0.0680 (19)	0.0369 (13)	−0.0017 (16)	0.0145 (12)	−0.0089 (13)
C45	0.0728 (16)	0.0550 (17)	0.0384 (13)	0.0037 (15)	0.0199 (12)	0.0079 (12)
C46	0.0648 (15)	0.0410 (14)	0.0382 (13)	−0.0020 (12)	0.0201 (12)	0.0007 (11)
F60	0.0584 (9)	0.1362 (17)	0.0781 (11)	0.0356 (10)	0.0216 (8)	−0.0059 (10)
C101	0.082 (3)	0.150 (5)	0.139 (4)	−0.012 (4)	0.016 (3)	−0.051 (4)
C102	0.114 (5)	0.185 (7)	0.110 (3)	−0.045 (4)	0.050 (3)	−0.003 (4)
C103	0.122 (3)	0.097 (3)	0.106 (3)	−0.032 (4)	−0.013 (3)	0.022 (3)

### Geometric parameters (Å, °)

**Table:**

N1—C9A	1.376 (3)	C15—H15	0.9300
N1—N2	1.378 (2)	C16—H16	0.9300
N1—C11	1.421 (3)	C31—C32	1.385 (3)
N2—C3	1.320 (3)	C31—C36	1.386 (3)
C3—C3A	1.443 (3)	C32—C33	1.381 (3)
C3—C31	1.478 (3)	C32—H32	0.9300
C3A—C4	1.390 (3)	C33—C34	1.374 (3)
C3A—C9A	1.425 (3)	C33—H33	0.9300
C4—C4A	1.424 (3)	C34—C35	1.361 (4)
C4—C41	1.495 (3)	C34—H34	0.9300
C4A—C5	1.423 (3)	C35—C36	1.382 (3)
C4A—C8A	1.429 (3)	C35—H35	0.9300
C5—C6	1.347 (3)	C36—H36	0.9300
C5—H5	0.9300	C41—C42	1.381 (3)
C6—F60	1.364 (3)	C41—C46	1.386 (3)
C6—C7	1.393 (3)	C42—C43	1.383 (3)
C7—C8	1.351 (3)	C42—H42	0.9300
C7—H7	0.9300	C43—C44	1.377 (3)
C8—C8A	1.421 (3)	C43—H43	0.9300
C8—H8	0.9300	C44—C45	1.369 (3)
C8A—N9	1.363 (3)	C44—H44	0.9300
N9—C9A	1.319 (3)	C45—C46	1.380 (3)
C11—C16	1.376 (3)	C45—H45	0.9300
C11—C12	1.378 (3)	C46—H46	0.9300
C12—C13	1.379 (3)	C101—C103^i^	1.362 (11)
C12—H12	0.9300	C101—C102	1.364 (13)
C13—C14	1.362 (4)	C101—H101	0.9300
C13—H13	0.9300	C102—C103	1.371 (12)
C14—C15	1.373 (4)	C102—H102	0.9300
C14—H14	0.9300	C103—C101^i^	1.362 (11)
C15—C16	1.377 (3)	C103—H103	0.9300
			
C9A—N1—N2	110.52 (16)	C16—C15—H15	119.5
C9A—N1—C11	130.36 (17)	C11—C16—C15	119.1 (2)
N2—N1—C11	119.06 (17)	C11—C16—H16	120.5
C3—N2—N1	107.82 (16)	C15—C16—H16	120.5
N2—C3—C3A	110.56 (17)	C32—C31—C36	118.7 (2)
N2—C3—C31	118.28 (18)	C32—C31—C3	119.97 (19)
C3A—C3—C31	131.16 (19)	C36—C31—C3	121.3 (2)
C4—C3A—C9A	118.28 (18)	C33—C32—C31	120.2 (2)
C4—C3A—C3	137.38 (19)	C33—C32—H32	119.9
C9A—C3A—C3	104.28 (17)	C31—C32—H32	119.9
C3A—C4—C4A	116.22 (18)	C34—C33—C32	120.3 (2)
C3A—C4—C41	122.66 (18)	C34—C33—H33	119.9
C4A—C4—C41	121.12 (18)	C32—C33—H33	119.9
C4—C4A—C5	121.79 (19)	C35—C34—C33	120.0 (2)
C4—C4A—C8A	119.85 (18)	C35—C34—H34	120.0
C5—C4A—C8A	118.31 (19)	C33—C34—H34	120.0
C6—C5—C4A	119.1 (2)	C34—C35—C36	120.4 (2)
C6—C5—H5	120.4	C34—C35—H35	119.8
C4A—C5—H5	120.4	C36—C35—H35	119.8
C5—C6—F60	118.6 (2)	C35—C36—C31	120.3 (2)
C5—C6—C7	124.0 (2)	C35—C36—H36	119.8
F60—C6—C7	117.5 (2)	C31—C36—H36	119.8
C8—C7—C6	118.3 (2)	C42—C41—C46	119.2 (2)
C8—C7—H7	120.9	C42—C41—C4	120.70 (19)
C6—C7—H7	120.9	C46—C41—C4	120.0 (2)
C7—C8—C8A	121.7 (2)	C41—C42—C43	119.9 (2)
C7—C8—H8	119.2	C41—C42—H42	120.0
C8A—C8—H8	119.2	C43—C42—H42	120.0
N9—C8A—C8	117.47 (19)	C44—C43—C42	120.3 (2)
N9—C8A—C4A	123.85 (19)	C44—C43—H43	119.9
C8—C8A—C4A	118.66 (19)	C42—C43—H43	119.9
C9A—N9—C8A	113.91 (17)	C45—C44—C43	120.0 (2)
N9—C9A—N1	125.35 (18)	C45—C44—H44	120.0
N9—C9A—C3A	127.85 (19)	C43—C44—H44	120.0
N1—C9A—C3A	106.80 (17)	C44—C45—C46	120.0 (2)
C16—C11—C12	120.3 (2)	C44—C45—H45	120.0
C16—C11—N1	119.84 (19)	C46—C45—H45	120.0
C12—C11—N1	119.84 (19)	C45—C46—C41	120.5 (2)
C11—C12—C13	119.4 (2)	C45—C46—H46	119.8
C11—C12—H12	120.3	C41—C46—H46	119.8
C13—C12—H12	120.3	C103^i^—C101—C102	118.7 (4)
C14—C13—C12	120.9 (2)	C103^i^—C101—H101	120.7
C14—C13—H13	119.6	C102—C101—H101	120.7
C12—C13—H13	119.6	C101—C102—C103	120.8 (4)
C13—C14—C15	119.3 (2)	C101—C102—H102	119.6
C13—C14—H14	120.4	C103—C102—H102	119.6
C15—C14—H14	120.4	C101^i^—C103—C102	120.5 (4)
C14—C15—C16	121.0 (2)	C101^i^—C103—H103	119.7
C14—C15—H15	119.5	C102—C103—H103	119.7
			
C9A—N1—N2—C3	0.8 (2)	C3—C3A—C9A—N1	1.2 (2)
C11—N1—N2—C3	178.12 (19)	C9A—N1—C11—C16	146.7 (2)
N1—N2—C3—C3A	0.1 (2)	N2—N1—C11—C16	−30.1 (3)
N1—N2—C3—C31	180.00 (18)	C9A—N1—C11—C12	−33.1 (3)
N2—C3—C3A—C4	176.2 (2)	N2—N1—C11—C12	150.1 (2)
C31—C3—C3A—C4	−3.7 (4)	C16—C11—C12—C13	−1.5 (4)
N2—C3—C3A—C9A	−0.8 (2)	N1—C11—C12—C13	178.3 (2)
C31—C3—C3A—C9A	179.3 (2)	C11—C12—C13—C14	1.6 (4)
C9A—C3A—C4—C4A	−1.6 (3)	C12—C13—C14—C15	−0.3 (4)
C3—C3A—C4—C4A	−178.4 (2)	C13—C14—C15—C16	−1.1 (5)
C9A—C3A—C4—C41	177.59 (19)	C12—C11—C16—C15	0.1 (4)
C3—C3A—C4—C41	0.8 (4)	N1—C11—C16—C15	−179.7 (2)
C3A—C4—C4A—C5	177.4 (2)	C14—C15—C16—C11	1.2 (4)
C41—C4—C4A—C5	−1.8 (3)	N2—C3—C31—C32	−41.7 (3)
C3A—C4—C4A—C8A	0.0 (3)	C3A—C3—C31—C32	138.2 (2)
C41—C4—C4A—C8A	−179.23 (19)	N2—C3—C31—C36	136.7 (2)
C4—C4A—C5—C6	−178.1 (2)	C3A—C3—C31—C36	−43.4 (3)
C8A—C4A—C5—C6	−0.7 (3)	C36—C31—C32—C33	−0.7 (3)
C4A—C5—C6—F60	179.0 (2)	C3—C31—C32—C33	177.8 (2)
C4A—C5—C6—C7	0.1 (4)	C31—C32—C33—C34	1.1 (4)
C5—C6—C7—C8	−0.2 (4)	C32—C33—C34—C35	−0.4 (4)
F60—C6—C7—C8	−179.1 (2)	C33—C34—C35—C36	−0.7 (4)
C6—C7—C8—C8A	1.0 (4)	C34—C35—C36—C31	1.1 (4)
C7—C8—C8A—N9	176.5 (2)	C32—C31—C36—C35	−0.4 (3)
C7—C8—C8A—C4A	−1.6 (4)	C3—C31—C36—C35	−178.8 (2)
C4—C4A—C8A—N9	0.9 (3)	C3A—C4—C41—C42	−66.5 (3)
C5—C4A—C8A—N9	−176.6 (2)	C4A—C4—C41—C42	112.7 (2)
C4—C4A—C8A—C8	178.9 (2)	C3A—C4—C41—C46	111.8 (2)
C5—C4A—C8A—C8	1.4 (3)	C4A—C4—C41—C46	−69.0 (3)
C8—C8A—N9—C9A	−178.0 (2)	C46—C41—C42—C43	−1.1 (3)
C4A—C8A—N9—C9A	0.0 (3)	C4—C41—C42—C43	177.2 (2)
C8A—N9—C9A—N1	177.3 (2)	C41—C42—C43—C44	−0.7 (4)
C8A—N9—C9A—C3A	−1.9 (3)	C42—C43—C44—C45	1.7 (4)
N2—N1—C9A—N9	179.3 (2)	C43—C44—C45—C46	−0.8 (4)
C11—N1—C9A—N9	2.4 (4)	C44—C45—C46—C41	−1.1 (4)
N2—N1—C9A—C3A	−1.3 (2)	C42—C41—C46—C45	2.1 (3)
C11—N1—C9A—C3A	−178.3 (2)	C4—C41—C46—C45	−176.3 (2)
C4—C3A—C9A—N9	2.9 (3)	C103^i^—C101—C102—C103	−1.0 (8)
C3—C3A—C9A—N9	−179.4 (2)	C101—C102—C103—C101^i^	1.0 (8)
C4—C3A—C9A—N1	−176.48 (18)		

Symmetry codes: (i) −*x*+2, −*y*, −*z*+2.

### Hydrogen-bond geometry (Å, °)

**Table:**

*D*—H···*A*	*D*—H	H···*A*	*D*···*A*	*D*—H···*A*
C16—H16···N2	0.93	2.58	2.844 (3)	97
C12—H12···N9	0.93	2.54	3.045 (3)	114
C44—H44···F60^ii^	0.93	2.58	3.374 (3)	143

Symmetry codes: (ii) −*x*+2, *y*+1/2, −*z*+3/2.
